# Volume Four

**Published:** 1864-08

**Authors:** 


### EDITORIAL DEPARTMENT.

### VOLUME FOUR.

Volume four dawns upon our editorial vision with more misgiving than ever; not that we are alarmed at the increased cost of publication, have any fears of our regular appearance, or ultimate success. We can rely upon the profession for pecuniary support, and the permanency of our "*institution*" is established—it was not "born to die." Our apprehension grows out of restriction of time for editorial labor, and a fear that it will be imperfectly performed. Few of our readers are aware of the time necessarily required in the conduct of our journal, or that this is extracted from hours usually devoted to sleep, with the little additional which is snatched in momentary fragments from active, constant professional employment. Some of our worthy contributors are ignorant of the care which is required to get manuscript through the press correctly, and wonder that typographical errors are not avoided. They do not know that the type is set by men ignorant of the professional language, and depending upon the letters for the word; that when it is all right, it is liable to be misplaced, and that corrections are some times almost unavoidably neglected. Those who have corrected their own proof have learned to expect errors, while those who do not understand the processes of publication can hardly understand the sources or error.

Again a little observation will show that any deficiency in the supply of original copy must often be made up by the editor, certainly if not furnished by others. It is necessity with him, to have copy, and it has always been the Buffalo medical editorial experience, that most of the city physicians are little to be depended upon for this sort of material, while fortunately, those in the towns and villages are comparatively more ready to furnish the results of their experience.

We have public institutions, hospitals, asylums, etc., attended by ambitious medical men, and full of material for observation and experience; we have physicians of extensive private practice, highly educated and unsurpassed in activity, and still volumes of the Journal are published and not an original article presented from most of these public or private sources; there is a great amount of clinical experience in Buffalo, but it cannot be furnished for the pages of the Journal, without time and thought in its preparation. It is not to be concealed, that many of the members of our profession depend largely for their laurels upon the amount of their professional incomes, and pay but little, very little attention to the cultivation of the science, either for their own or others benefit; and on this account the effort to sustain a medical journal requires to be much greater than if all were ambitious of contributing to the sum of medical knowledge. Notwithstanding all these discouragements the pages of the Journal have never "gone empty away," and it is believed has been unsurpassed in the variety and practical value of its contents.

We most cordially invite original articles for our pages, and again remind our readers that the Journal is published in the interest of the whole profession, and depends only upon it for countenance and support. If each subscriber will furnish a carefully prepared paper upon some medical subject, and influence at least one additional subscriber for our Journal, we shall then be able to furnish such a medical publication as is found no where else.

We have spoken of what should be expected of the members of the profession, as a sort of wholesome stimulus, and not by way of complaint; for we feel under the deepest obligations of gratitude and have reason for thankfulness beyond expression. We have received encouragement and support far beyond any expectation, and shall strive to make the Journal in future still more valuable and worthy of patronage.

We have been hoping to continue without advance in price of subscription, and have made every reasonable effort to avoid it. It needs no explanation; publication expenses have doubled since our last year's contract, and publishers have fulfilled their agreement at considerable loss. We most reluctantly submit to a necessity which is universal with publishers, but which we think cannot long continue. We are obliged to place the price of volume four at three dollars, but we have made arrangements by which any depreciation in the price of paper or labor shall be added to the number of pages, by which means we hope before many months to be able to increase the size of our Journal so as to furnish a reprint of every valuable paper upon the science, or improvement in the practice of medicine, published either in American or foreign journals. If we can increase the number of our pages to sixty-four we shall have accomplished a long cherished object of ambition, and shall be able to furnish our readers with a full monthly *resume* of the advance of medical and surgical science. Advance in price of all the common articles of consumption, and consequent increase in the value of labor, would not have driven us from our former prices, but "*cotton is king*" in this matter, more truly than we had supposed, and is levying upon us heavy imposts; however we shall furnish the Journal at the very lowest possible price of publication, and trust that our patrons will assist us in meeting the unavoidable necessity.

We anticipate a greatly improved volume, and have made arrangements by which we hope to furnish the original department with a variety of valuable articles upon practical subjects. We shall be able to continue, through the kindness of the Secretary, Dr. Peters, regular reports of the proceedings of the Buffalo Medical Association, and Hospital and Clinical reports will also continue as a feature of the new volume. The other departments of the Journal will be as full and varied as space will permit. Hoping for co-operation and support, we gird ourselves for yet greater exertion.

